# A novel and efficient method for producing high-purity single protoplast-derived isolates of *Plasmodiophora brassicae*

**DOI:** 10.3389/fmicb.2026.1789807

**Published:** 2026-04-13

**Authors:** Ling Cao, Qilin Chen, Jinghe Wang, Hao Hu, Fengqun Yu

**Affiliations:** Saskatoon Research and Development Centre, Agriculture and Agri-Food Canada, Saskatoon, SK, Canada

**Keywords:** *Plasmodiophora brassicae*, race characterization, single-nucleotide polymorphisms, single-protoplast-derived isolates, whole-genome sequencing

## Abstract

*Plasmodiophora brassicae*, a root-infecting protist, causes the devastating clubroot disease of cruciferous crops worldwide. Field populations of this pathogen often consist of multiple strains, leading to inconsistent experimental results and unreliable resistance classification in breeding programs. The extremely low infection success rate associated with single resting spore inoculation has limited the generation of genetically uniform single spore-derived isolates (SSIs). In this study, we developed a simple and highly effective single-protoplast-derived isolate (SPI) method to obtain near-genetically pure isolates of *P. brassicae*. The method involves enzymatic digestion of clubroot-infected root tissue to remove plant cell wall and release individual plant protoplasts. Each released protoplast contains thousands of resting spores. Individual protoplasts are then isolated and purified from the digestion suspension. All resting spores contained within a single protoplast are subsequently released and used as the sole inoculum to generate one SPI on susceptible canola seedlings. Using soilless Sunshine Mix #3, SPI infection success rates ranged from 77.8 to 100%, depending on the strain. Genetic purity of 20 SPIs derived from strain AB11 was assessed using Kompetitive Allele-Specific PCR, revealing 95% homogeneity at informative SNP loci. Whole-genome sequencing further demonstrated a marked reduction in genomic heterogeneity, from 91.22 to 96.40% in the progenitor strains AB11 and AB16, respectively, to ≤ 1.0% in their corresponding SPIs, compared with 14.95 and 44.26% observed in two of nine published SSIs. Using a panel of *Brassica napus* lines, each carrying a single clubroot resistance gene (*Rcr1*, *Rcr3*, *Rcr5*, *Rcr8*, *Rcr9*, or *Rcr10*), we identified the corresponding avirulence (*Avr*) genes among 18 SPIs. The frequencies of *Avr1*, *Avr3*, *Avr5*, *Avr8*, *Avr9*, and *Avr10* were 0.0, 11.1, 44.4, 33.3, 27.8, and 50.0%, respectively. Seven races were identified, including the highly aggressive race *avr1-3-5-8-9-10*, underscoring the urgent need for novel resistance genes. The SPI method provides a robust, efficient, and reproducible strategy for generating near–genetically pure *P. brassicae* isolates with high infection success, improving the reliability of resistance screening and virulence monitoring, and facilitating informed and durable deployment of clubroot resistance genes for long-term disease management.

## 1 Introduction

*Plasmodiophora brassicae* Woronin, an obligate biotrophic, soil-borne pathogen, causes devastating clubroot disease in *Brassicaceae* crops worldwide, including canola, cabbage, and broccoli, resulting in significant yield losses and economic damage (Dixon, 2009; Rempel et al., 2014; Botero-Ramírez et al., 2022; Lai et al., 2025). The pathogen induces abnormal root tissue growth, forming characteristic “clubs” that disrupt water and nutrient transport (Ingram and Tommerup, 1972; Kageyama and Asano, 2009; Tu, 2018; Botero-Ramírez et al., 2022; Lai et al., 2025). Its resting spores exhibit remarkable longevity, remaining viable in soil for up to 18 years and resuming pathogenicity upon encountering a susceptible host (Macfarlane and Last, 1959; Wallenhammar, 1996; Wallenhammar, 1999; Peng et al., 2015; Saharan et al., 2021). Therefore, robust clubroot disease management is crucial to mitigate its persistent threat to *Brassicaceae* crops. To date, developing resistant cultivars is the most effective and environmentally sustainable strategy for managing clubroot. Numerous clubroot resistance (*CR*) genes and quantitative trait loci (QTLs) have been identified across Brassica genomes (Kuginuki et al., 1997; Hirai et al., 2004; Piao et al., 2009; Ueno et al., 2012; Hatakeyama et al., 2013; Pang et al., 2014; Yu et al., 2016; Huang et al., 2017; Niemann et al., 2017; Yu et al., 2017; Nguyen et al., 2018; Chang et al., 2019; Karim et al., 2020; Yu et al., 2021; Rahaman et al., 2022; Zhang et al., 2022), allowing the development of resistance cultivars of *Brassica* crops through conventional breeding (Frauen, 1999; Diederichsen et al., 2006; Kawasaki et al., 2021; Yu et al., 2021; Kaur et al., 2022; Song et al., 2022; Zhan et al., 2022), or advanced biotechnological approaches, such as a modified CRISPR/Cas9-based cis-genic vector system (Hu and Yu, 2024; Hu et al., 2024; Masud Karim and Yu, 2024).

In light of the gene-for-gene theory (Flor, 1956), a specific plant resistance gene encodes a protein that recognizes a corresponding avirulence (*Avr*) gene product from the pathogen, triggering a strong immune response to prevent further infection. Considering the genetic diversity found in clubroot pathogen samples, sets of differential *Brassica* hosts are currently utilized to classify pathogens into pathotypes (Buczacki et al., 1975; Donald et al., 2006). However, *P. brassicae* exists as mixtures of pathotypes in soil, and single galls from Alberta canola fields often contain multiple strains (Fu et al., 2020), indicating the need for cautious interpretation of results when the mixture of the population is subject to pathotyping or being used as an inoculum in plant pathology research. Importantly, resistance tests from mixed pathotypes in breeding programs showed poor reproducibility (Diederichsen et al., 2016), potentially leading to inaccurate resistance labeling and impeding the development of resistant cultivars.

To accurately identify *CR* genes, obtaining genetically uniform *P. brassicae* isolates is critical but particularly challenging compared to other fungal pathogens or bacteria, as this pathogen cannot be cultured *in vitro*. Many research laboratories have made tremendous efforts to generate single resting spore-derived isolates (SSIs) to purify the pathogen genotypes, typically by inoculating susceptible hosts with single resting spores (Buczacki, 1977; Tinggal and Webster, 1981; Jones et al., 1982; Scott, 1985; Kageyama et al., 1995; Narisawa et al., 1996; Xue et al., 2008; Askarian et al., 2021a; Askarian et al., 2021b). Buczacki first reported the successful generation of SSIs using this single-spore inoculation approach (Buczacki, 1977). However, the extremely low infection success rate of single resting spores greatly limited the broader application of this technique. Earlier strategies to improve efficiency included inoculating with large resting spores or with root hairs presumed to contain a single sporangium to obtain genetically uniform isolates. However, infection rates generally remained low—only 1.3% using single large resting spores (Scott, 1985), up to 13.25% on 2-week-old seedlings for pathogen SACAN03-1 (Xue et al., 2008), and 0–17.9% with root hairs, depending on the strain (Diederichsen et al., 2016). To reduce contamination from additional spores, some studies isolated and verified single resting spores microscopically rather than relying on dilution of a spore suspension, (Jones et al., 1982; Scott, 1985). More recently, trapping single spores in agar has been introduced to improve spore–plant contact (Lv et al., 2021).

The low success rate of single spore inoculation likely results from the extremely limited number of spores used (only one), coupled with the challenges in resting spore germination, the critical first step in infection. Spore germination is influenced by numerous environmental variables, including soil moisture, pH, temperature, ion concentrations, and other biotic and abiotic factors, all of which vary under different circumstances (Macfarlane, 1970; Friberg et al., 2005; Rashid et al., 2013; Auer and Ludwig-Müller, 2023; Wang et al., 2023; Kang et al., 2024; Ghimire et al., 2025). To date, the specific combination of factors required to reliably trigger resting spore germination remains unknown, making it impossible to optimize conditions for consistent single spore infection. Overall, SSI generation is both labor-intensive and time-consuming (Diederichsen et al., 2016).

To overcome these limitations, we developed a novel and efficient method for generating high-purity single-protoplast-derived isolates (SPIs) of *P. brassicae*. This approach exploits the natural compartmentalization of the pathogen within individual infected cortical cells. By isolating a single root protoplast and using all contained resting spores as the inoculum, the SPI method achieves near-single-genotype purity while increasing infection success rates to 77.8–100%. The genetic homogeneity of these isolates was rigorously validated using SNP genotyping and whole-genome sequencing, which revealed heterogeneity levels of ≤ 1%—comparable to or lower than those of traditional SSIs. Consequently, the SPI method provides a practical, reproducible, and scalable platform for pathogen purification, enabling accurate race characterization, reliable *Avr* gene assignment, and the strategic deployment of clubroot resistance genes in *Brassica* breeding programs.

## 2 Materials and methods

### 2.1 Plant material and plant growth conditions

A clubroot-susceptible DH line derived from the canola cultivar “Westar,’ DHW, was used for propagating *P. brassicae* strains and SPIs, and generating SPIs. CR lines and DH16516 (a susceptible control) were utilized to evaluate the virulence of *P. brassicae* strains and SPIs. Seeds were germinated for 2 days in 4-inch square pots filled with a soilless mix (Sunshine Mix #3, Sun Gro^®^ Horticulture Canada Ltd) amended prior to potting with 1,333 g of Osmocote (Everris NA Inc.; Dublin, OH, United States) per 3.8 cu.ft., an maintained in a growth chamber (Conviron, Winnipeg, Manitoba, Canada) at 20°C/18°C (day/night) under a 16/8 h (light/dark) photoperiod. Seedlings were watered before inoculation and covered with a transparent dome for 1 week afterward to maintain soil moisture.

### 2.2 *P. brassicae* strains and inoculation

Strains of *P. brassicae* collected from canola fields in Alberta (AB) and Saskatchewan (SK), as detailed in Supplementary Table 4, were used in this study. These field isolates were kindly provided by Dr. Stephen Strelkov (University of Alberta, Canada), Ms. Coreen Franke (Nutrien Ag Solutions, Saskatoon, SK, Canada), Dr. Godfrey Chongo (BASF Canada Inc., Saskatoon, SK, Canada), and Drs. Barbara Ziesman and Alireza Akhavan (Government of Saskatchewan, Canada).

Six-day-old DHW seedlings were each inoculated with 2 mL of a resting spore suspension at a concentration of 1 × 10^7^ spores/mL. Fresh galls were harvested 5 weeks post-inoculation for protoplast isolation or genomic DNA extraction, or stored at –20°C for future use.

### 2.3 Purification of *P. brassicae* resting spores

Six grams of *P. brassicae*-infected canola roots were homogenized in the WARING blender with 50 mL sterile water at high speed for 2 min and filtered through two layers of MIRACLOTH (Merck KGaA Darmstadt, Germany). The filtrate was then centrifuged in 50-mL centrifuge tubes at 3,000 rpm for 15 min to collect the resting spores. Based the principle of sucrose density gradient separation (Britten and Roberts, 1960), the spore pellet was resuspended in 10 mL of 50% sucrose (w/v) and centrifuged at 2,000 rpm for 15 min to remove heavier particles, such as soil, large debris, or polysaccharides, which settled at the bottom of the tube, while the resting spores remained in the 50% sucrose layer. The suspension was carefully transferred to a new 50 mL tube without disturbing the white polysaccharide pellet and brown soil/debris pellet at the bottom. The volume was adjusted to 50 mL with sterile distilled water (dH_2_O) (final sucrose concentration ∼10%), and the suspension was centrifuged at 3,000 rpm for 15 min to collect the resting spores, while the smaller particles remained in the 10% sucrose layer. The supernatant was discarded. This two-step sucrose purification (50% → 10%) was repeated once to further improve purity. The final spore pellet was resuspended in sterile dH_2_O and washed twice by repeated resuspension and centrifugation. The purified spores were then aliquoted into 1.5 mL microcentrifuge tubes, pelleted by centrifugation at 14,000 rpm for 2 min, and the supernatant was discarded. Purified resting spores were either stored at -20°C or used immediately for genomic DNA extraction or other downstream applications.

### 2.4 Isolation and purification of clubroot-infected root protoplasts

**FIGURE 1:**
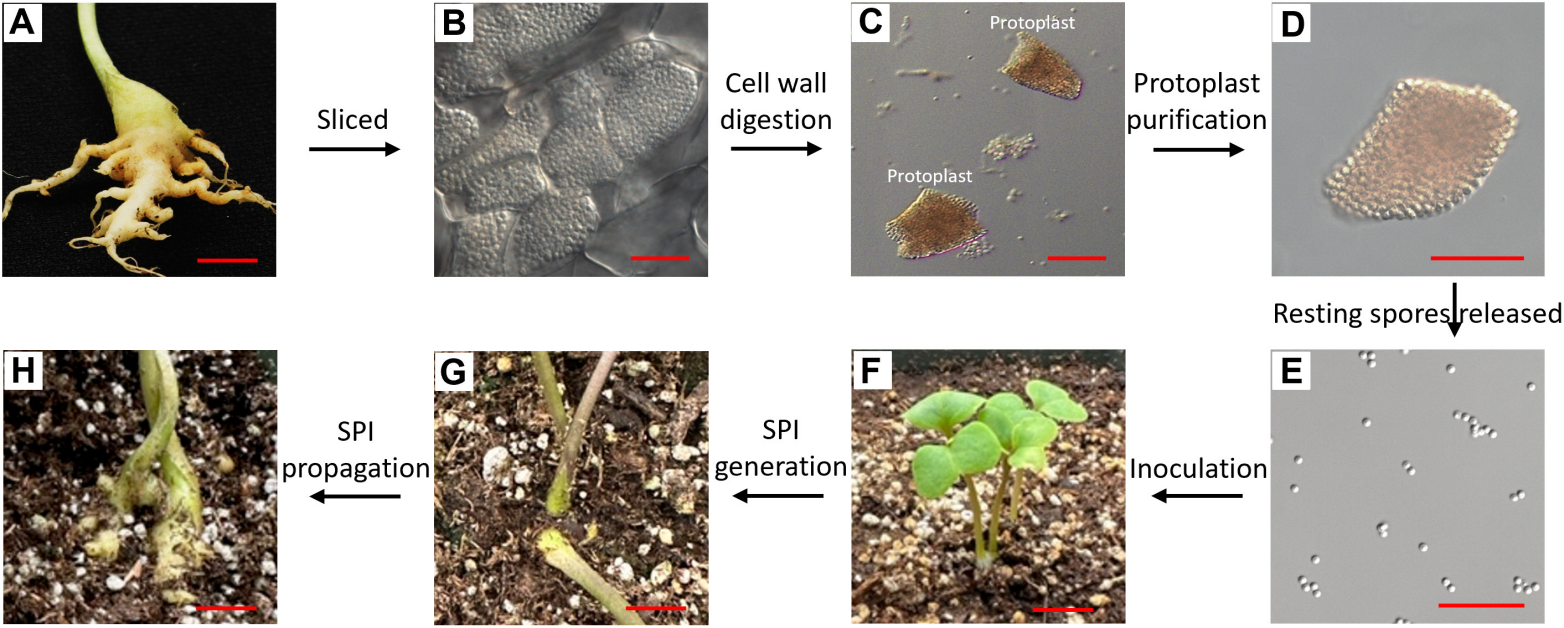
Procedures for developing genetically pure isolates of *P. brassicae* by isolating single root protoplasts to derive isolates. A *P. brassicae* strain, AB11, was selected to demonstrate the generation of single protoplast-derived isolates (SPIs). **(A)** Fresh gall was used for isolating of plant protoplasts. **(B)** Longitudinal section of the clubbed root indicating root cells were filled with spores. **(C)** Protoplasts were released after cell wall digestion of infected roots. **(D)** An intact protoplast filled with spores was selected and transferred under an inverted microscope. **(E)** Resting spores was released from the selected intact protoplast. **(F)** All three seedlings in one pot were inoculated with resting spores from one protoplast. **(G)** Visible galls emerged 3 weeks after inoculation with resting spores from a single protoplast, indicating the successful generation of a single-protoplast isolate (SPI). **(H)** Propagation of original SPIs. Resting spores from an original SPI were collected and used to inoculate susceptible hosts to propagate the SPI. Scale bars in **(A,F–H),** and **(B–E)** equal 10 mm and 20 μm, respectively.

A large, freshly induced gall produced by individual field strains (e.g., AB11) at 5 wpi was harvested for protoplast isolation (Figure 1A). Microscopic examination of root sections under a DIC microscope revealed cortical cells densely packed with mature spores (Figure 1B). Cellulase (C-0901, Sigma-Aldrich, United States) and pectolyase (P3026, Sigma-Aldrich, United States) were used to enzymatically digest cellulose and pectin in the root cell walls, facilitating efficient protoplast release. These enzymes target plant cell wall components and are not expected to affect the integrity and viability of *P. brassicae* resting spores. The gall was cut into small pieces using a scalpel or razor blade and transferred to a 60 × 15 mm Petri dish containing 4 mL of Plant Protoplast Digest/Wash solution (D9692, Sigma-Aldrich, United States) supplemented with 1.0% cellulose and 0.5% pectinase. Samples were incubated in the dark at ambient laboratory temperature (22–23°C) on a shaker at 40 rpm for 1.5 h to facilitate cell wall digestion (Yao et al., 2016).

Enzymatic removal of the cell walls from gall tissue released both protoplasts (intact and ruptured) and free resting spores liberated from ruptured cells (Figure 1C). Intact protoplasts were individually selected under an inverted microscope (OLYMPUS CKX53) with a 20 × lens, and transferred with a 10 μL pipette tip and a corresponding pipette into a clean single-cavity slide containing 100 μL of distilled water. This purification step was repeated until a single protoplast could be aspirated with no contaminating free resting spores visible within the microscope field of view (Figure 1D). The purified protoplast was then transferred into a 1.5 mL Eppendorf tube pre-filled with 100 μL of distilled water (dH_2_O).

### 2.5 Generation of *P. brassicae* SPIs

Forty small beads from the Lysing Matrix A tubes (MP Biomedicals) were added to the tube containing a single protoplast in 100 μL of dH2O, which was then vortexed at low speed for 12 min using a Multi-Tube Vortex Mixer to rupture the protoplast and release the resting spores (Figure 1E). The resulting 100 μL spore suspension was used to inoculate 3, 6-day-old DHW seedlings, in order to increase the possibility of successful infection in a single pot (Figure 1F). Disease severity was evaluated 5 weeks post-inoculation. If any of the three DHW plants exhibited symptoms rated as 2 or 3 on the scale described by Kuginuki et al. (1999), the infection was considered successful and classified as producing an SPI (Figure 1G). Galls from each SPI were then collected for propagation on the susceptible DHW (Figure 1H), genomic analysis, or further experiments.

### 2.6 Genomic DNA extraction

DNA was extracted using a modified cetyltrimethylammonium bromide (CTAB) method (Allen et al., 2006). The purified resting spores were lyophilized in a LABCONCO™ Freeze Dryer, transferred into Lysing matrix A tubes, and ground for 2 min with an MP FastPrep-24 ^®^ homogenizer. Each tube was added 600 μL of CTAB buffer (2% CTAB, 10 mM EDTA pH 8.0, 100 mM Tris-HCl pH 8.0, 1.4 M NaCl), briefly vortexed, and incubated at 65°C in a heating block for 30 min, followed by centrifugation at 12,000 rpm for 2 min. The supernatant was transferred to new tubes, mixed with 500 μL of chloroform, and centrifuged again to separate the phases. Approximately 350 μL of the upper aqueous phase was transferred to a new tube, and large molecular weight RNA was precipitated by adding half a volume of 1 M LiCl. After centrifugation at 12,000 rpm for 8 min, the supernatant was transferred to a new tube, and genomic DNA was precipitated by adding two volumes of 100% ethanol and 0.2 volume of 5 M NaCl. The DNA samples were then treated with RNase A (10 μg/mL) at 37°C for 40 min, then re-precipitated, washed with 70% ethanol, and resuspended in 200 μL of 1x TE buffer (10 mM Tris-HCl, 1 mM EDTA, pH 8.0). DNA quality was evaluated using a NanoDrop spectrophotometer, and concentration was determined with the Quant-iT™ PicoGreen ^®^ dsDNA assay on a BMG-LABTECH OMEGA reader.

### 2.7 Kompetitive Allele Specific PCR SNP genotyping marker development

The RADseq sequences of 21 clubroot pathogen strains (Supplementary Table 2) were downloaded from the Sequence Read Archive Home-SRA-NCBI and aligned to the reference genome pbe3.h15 (GenBank assembly GCA_001049375.1) using bwa-mem with default parameters for accurate short-read mapping (Li and Durbin, 2009; Li, 2013). DNA variants, including SNPs and InDels, were populated by bcftools (Altinkaya et al., 2025). To assess the genetic purity of clubroot isolates, 60 bi-allelic SNPs were randomly selected from 19 different contigs (Supplementary Table 3), and analyzed using KASP™ chemistry (LGC Biosearch Technologies) with primers designed according to Semagn et al. (2014), generating PCR products ranging from 80 to 120 base pairs (bp) (Supplementary Table 3).

### 2.8 Whole-genome sequencing

Whole-genome sequencing of the clubroot strains and their derived SPIs in this study was performed using Illumina technology. Libraries were prepared according to the manufacturer’s protocol using Illumina DNA Prep-Tagmentation (M) Beads, Illumina DNA Prep-IBP+Buffers, Illumina DNA Prep-PCR+Buffers, and Nextera DNA CD indexes. Briefly, genomic DNA was fragmented into smaller pieces, ligated with specialized adapters at both ends, and size-selected for fragments ranging from 300 to 1,000 bp. The selected fragments for each sample were then PCR-amplified and combined into a pooled library for sequencing. Sequencing was carried out on an Illumina NovaSeq platform using a paired-end 150 bp (PE150) flow cell.

### 2.9 Short reads assembly, SNP calling, and analysis

The next-generation sequencing data were analyzed using the software package Lasergene Genomics Suite 17 (DNASTAR, Madison, WI) as described by Chen et al. (2021). Paired-end short reads (150 bp) were first assembled to the genome scaffold pbe3.h15 (GenBank GCA_001049375.1) using SeqMan.

Following alignment, mapping statistics were obtained from the alignment summary report generated by the software. The read mapping rate was calculated as the proportion of total reads successfully aligned to the reference genome. Genome coverage was determined as the percentage of reference bases covered by at least one mapped read, and sequencing depth was calculated as the average read depth across the reference genome.

Variants (SNPs and InDels) were called by QSeq based on the following filtering criteria: P not ref ≥ 90%, Q call ≥ 25, minimum SNP percentage ≥ 90%, depth ≥ 10.^1^ Identified variants were exported and further filtered (Phred Q score > 25, MAF ≥ 5, depth ≥ 10) for subsequent SNP data mining. Genome-wide heterogeneous SNP percentages were analyzed, with strains or SPIs classified as genetically pure if the heterogeneity rate was close to or below 1%.

### 2.10 Identification of races of *P. brassicae* single-protoplast isolates

A set of spring-type *B. napus* single gene lines (SGLs), each carrying one of the six *CR* genes *Rcr1*, *Rcr3*, *Rcr5*, *Rcr8*, *Rcr9*, or *Rcr10*—was developed in our laboratory (Chu et al., 2014; Huang et al., 2017; Yu et al., 2017; Yu et al., 2021; Rahaman et al., 2022; Zhang et al., 2026). These lines were used to assess *P. brassicae* virulence patterns, with their recurrent parent canola line DH16516 included as a susceptible control. Ten seeds of each line were sown per pot, with two pots used as biological replicates. Seedlings were thinned to six per pot before inoculation. Six days after sowing, the seedlings were inoculated with 2 mL inoculum per plant at a concentration of 1 × 10^7^ spores/mL. The trays were covered with transparent plastic domes for 1 week to maintain humidity. Six weeks post inoculation, roots were harvested, cleaned, and evaluated for clubroot symptoms. Clubroot disease severity was assessed on a scale of level 0–3, where level 0 = no symptoms, level 1 = a few small clubs on lateral roots, level 2 = small size of clubs on primary root or large clubs on lateral roots, and level 3 = moderate or large size of clubs on primary root. This scale was modified based on Strelkov et al. (2006). A disease severity index (DSI) was calculated for each line using the following formula:


$$\text{DSI}=\frac{\sum \left(\mathrm{severity}\,\mathrm{level}\right)\times \left(\mathrm{number}\,\mathrm{of}\,\mathrm{plants}\,\mathrm{rated}\,\mathrm{at}\,\mathrm{the}\,\mathrm{level}\right)}{\left(\mathrm{total}\,\mathrm{number}\,\mathrm{of}\,\mathrm{plants}\,\mathrm{per}\,\mathrm{pot}\right)\times 3}\times 100$$

When DSIs differed between the two replicates, the higher DSI was considered more accurate and used as the response of SGLs to the SPI tested. A DSI threshold of 30% was established to distinguish between resistance and susceptibility: SGL lines with a DSI ≤ 30% were classified as resistant (R), while those with a DSI > 30% were classified as susceptible (S). Based on gene-for-gene theory, an avirulence (*Avr*) or virulence (*avr*) gene was assigned to each SPI, corresponding to an R-eliciting or S-eliciting reaction, respectively, and the combination of all *Avr* genes present in a SPI was defined as its race. The frequency of these *Avr* genes was also assessed within the pathogen populations.

## 3 Results

### 3.1 Optimal stage for clubroot-infected root protoplast isolation

**FIGURE 2:**
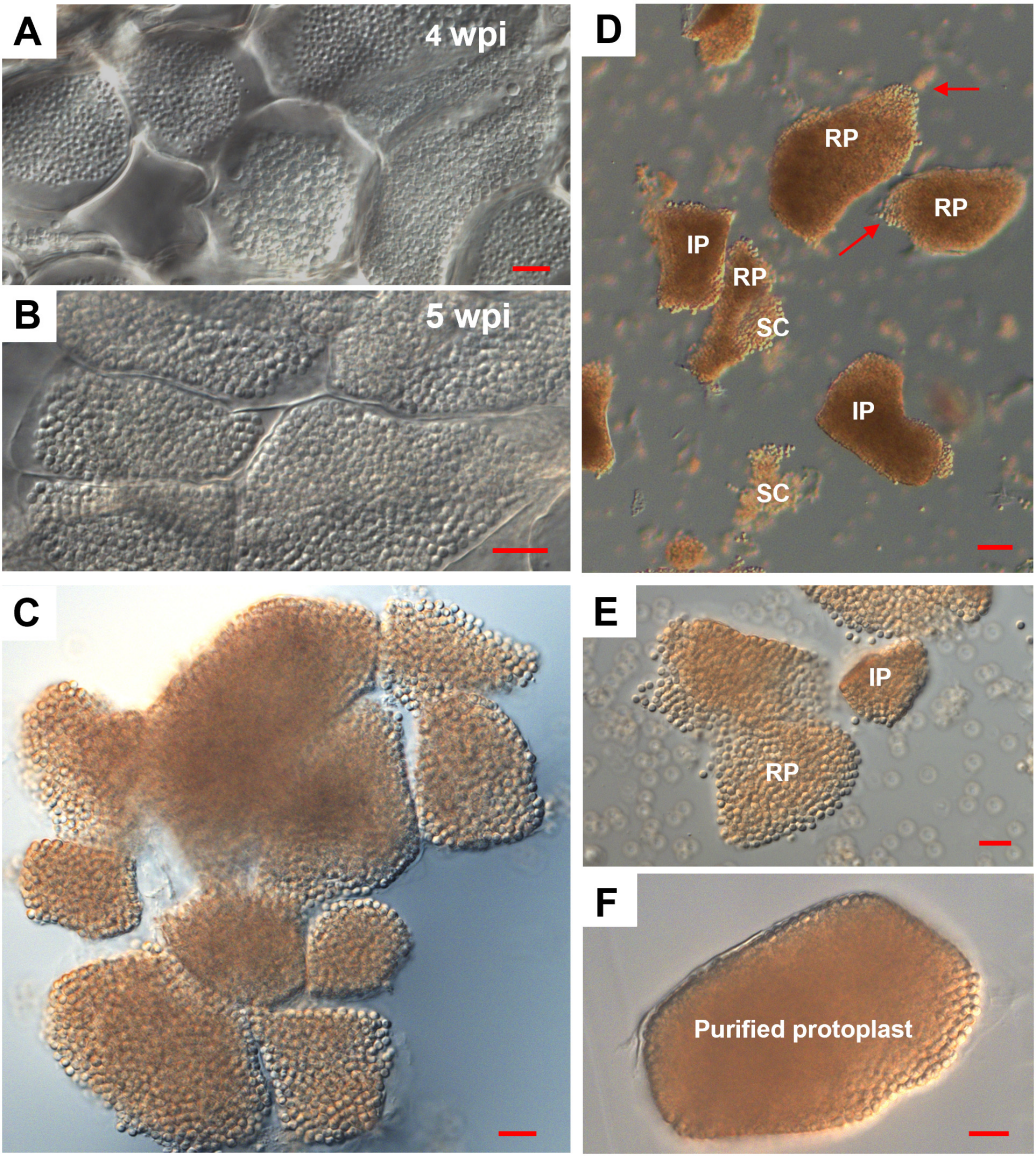
Morphology of protoplasts isolated from clubroot-infected roots. **(A,B)** Longitudinal sections of galls on DHW roots at 4 weeks (A) and 5 weeks **(B)** post-inoculation (wpi) with *P. brassicae* strain AB11. At 4 wpi, cortical cells contained immature resting spores; by 5 wpi, cells were densely packed with mature, condensed resting spores. Following enzymatic cell wall digestion of 5-wpi gall tissue, **(C)** incomplete digestion resulted in clusters of partially separated protoplasts with clearly visible boundaries between adjacent protoplasts. **(D)** After complete cell wall digestion, the suspension contained intact protoplasts (IP), ruptured protoplasts (RP) with loosely associated resting spores (the rupture site is indicated by a red arrow), spore clusters (SC), and free resting spores were observed under the microscope with a 20× lens. **(E)** With a 40× lens, loosely associated resting spores within a single ruptured protoplast and an adjacent small intact protoplast were observed. **(F)** The purified intact protoplast was examined with a 40× lens. Scale bars = 10 μm in **(A–F)**.

To determine the optimal stage for isolating protoplasts containing mature resting spores and to establish a reliable isolation protocol, galls were harvested from a highly susceptible doubled-haploid canola line DHW, a DH line developed from cultivar “Westar” at 4 and 5 wpi with a *P. brassicae* strain, AB15. Gall tissues were longitudinally sectioned, cleared, and examined under a Leica DM2500 microscope equipped with DIC optics (Cao et al., 2018). At 4 wpi, cortical cells contained predominantly immature resting spores, whereas at 5 wpi the cells were densely packed with condensed, mature resting spores (Figures 2A,B). Consequently, 5 wpi was selected as the optimal time point for protoplast isolation, enabling the recovery of all resting spores contained within one individual protoplast as a single inoculum to generate a single-protoplast-derived isolate (SPI).

### 3.2 Morphological characterization of protoplasts for selection

Intact protoplast selection is a critical step for producing high-purity SPIs of *P. brassicae.* Occasionally, incomplete cell wall digestion caused adjacent protoplasts to remain partially attached, forming protoplast clusters with clearly defined boundaries between neighboring protoplasts that were clearly visible (Figure 2C). After complete digestion, intact and ruptured protoplasts, spore clusters and free spores were observed in the resulting suspension (Figure 2D). Ruptured protoplasts released resting spores that were loosely associated at the rupture site and exhibited no visible boundaries among those resting spores (Figure 2E). Fully dissociated intact protoplasts displayed a sharply defined outer boundary, were densely packed with resting spores and retained the original shape and structural integrity of the infected root cortical cells, with no visible internal boundaries (Figure 2E). This morphology of the intact protoplasts contrasts sharply with protoplasts isolated from healthy, differentiated root cells, which are typically spherical and bounded solely by the plasma membrane due to the presence of a big central vacuole (Yao et al., 2016). Prior to natural spore release, the fibrous material surrounding the resting spores in intact protoplasts, as previously reported (Kageyama and Asano, 2009), likely confers slight resistance to mechanical disruption, thereby helping to preserve protoplast integrity during the isolation and transfer of individual protoplasts (Figure 2E). These protoplasts were purified (Figure 2F) and subsequently used throughout this study for the generation of SPIs.

### 3.3 Efficiency and success rate of SPI generation

**FIGURE 3:**
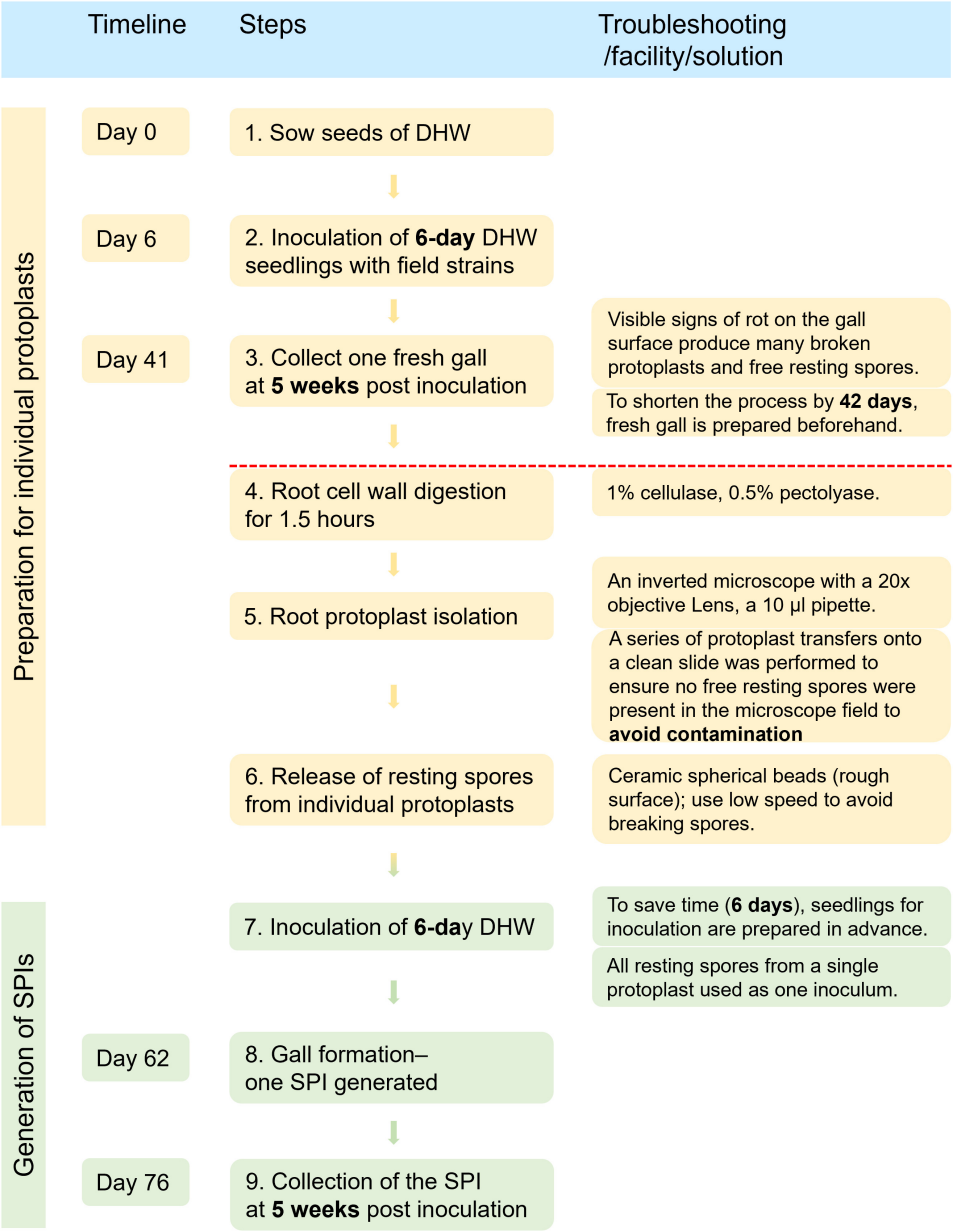
Timeline and steps for generating single protoplast-derived isolates (SPIs) of *P. brassicae*.

A detailed timeline and step-by-step workflow for generating single SPIs of *P. brassicae* are presented in Figure 3. The complete process required a total of 76 days. When fresh galls for protoplast isolation and 6-day-old DHW seedlings for inoculation are prepared in advance, the duration can be shortened to approximately 5 weeks, equivalent to one generation of clubroot propagation on canola in a growth chamber or greenhouse.

To quantify resting spores per root protoplast, the number of resting spores in 1 μL of the 100 μL inoculum derived from each of 12 single protoplasts was counted using a hemocytometer, revealing a range of 1,000–9,000 spores per protoplast (Supplementary Table 1). Conventionally, resting spore concentrations for inoculation often reach 10^7^ spores/mL to ensure consistent and successful infection. The number of resting spores from a single protoplast is significantly lower than the concentrations typically used in laboratories. To evaluate the success rate of generating SPIs, four strains from Alberta (AB) and two from Saskatchewan (SK) fields were tested (Table 1). Infection rates varied among the strains, ranging from 77.8 to 100% (Table 1).

**TABLE 1:** Success rate of generating *P. brassicae* single protoplast-derived isolates (SPIs).

Pb strains	No. of single protoplasts (SPs) for SPI generation	No. of SPIs generated	Success rate
AB06	9	7	77.8%
AB11	12	12	100.0%
AB15	7	7	100.0%
AB20	7	7	100.0%
SK01	12	12	100.0%
SK29	12	10	83.3%

Four strains from Alberta and two from Saskatchewan fields were selected for generating *P. brassicae* SPIs. Resting spores derived from single root protoplasts were used to inoculate the clubroot-susceptible doubled haploid (DH) line DHW seedlings and assess the success rate of generating SPIs.

### 3.4 Preliminary purity test of SPIs via single-nucleotide polymorphism genotyping

If allelic variations exist within single strains, corresponding bi-allelic SNP markers can be used to confirm the homogeneity of SPIs at those SNP loci through SNP genotyping. Sixty bi-allelic SNPs were randomly selected from 19 different contigs (Supplementary Table 2) for SNP marker development (Supplementary Table 3). The SNP markers were analyzed using KASP assays by genotyping sixteen strains collected from fields in Western Canada, as well as five SPIs and three negative controls (Supplementary Table 4). Only the SNPs that reveal heterogeneity in the strains are considered useful for verifying the purity of SPIs derived from these strains.

**FIGURE 4:**
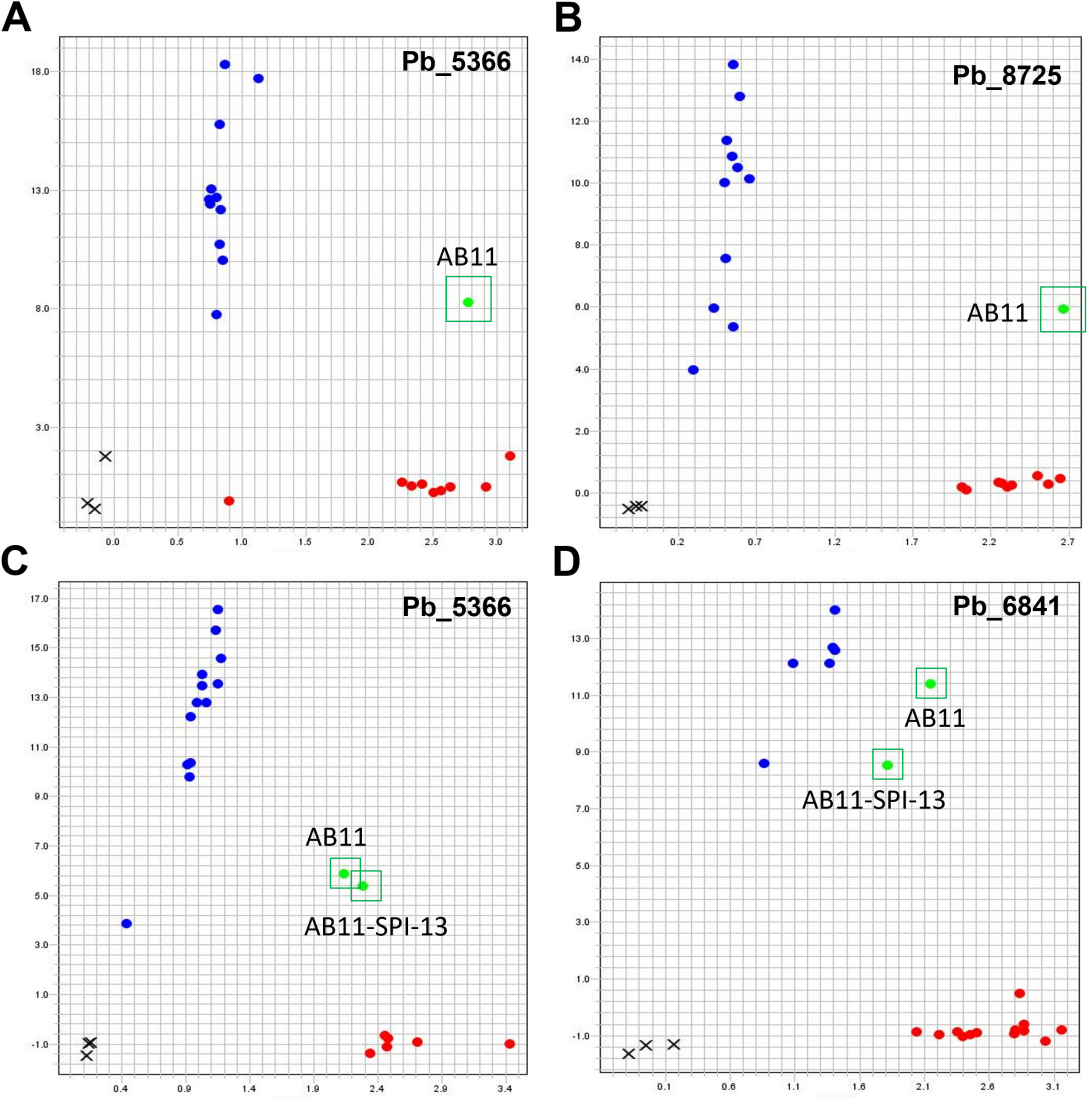
Identification of SNP markers for detection of genetic diversity and KASP analyses of AB11 single protoplast-derived isolates (SPIs). The SNP markers were investigated in a small population of sixteen *P. brassicae* strains, five SPIs derived from strain AB25 and three negative controls using KASP assay **(A,B)**. Both alleles were detected within the strain of AB11 at the DNA position of Pb_5366 **(A)** and Pb_8725 **(B)**. Twenty AB11 SPIs were genotyped by KASP SNP markers Pb_5366 **(C)** and Pb_6841 **(D)**, along with one progenitor strain AB11 and three negative controls. Six SPIs indicated one allele, thirteen exhibited the other allele, while the remaining one showed both alleles, same as the progenitor strain AB11.

Based on the number of distinctive clusters (Table 2 and Supplementary Figure 1) after interrogating SNPs in 21 strains/isolates, the KASP assays in this study were categorized into four groups. Group 1 did not generate any distinctive clusters. Ten SNP markers, such as Pb_7812 (Supplementary Figure 1A), were assigned to this group. Group 2 generated only one homogeneous cluster, along either *Y*- or *X*-axis. The group consisted of 15 SNP markers, such as Pb_8910, indicating monomorphism among the strains. Eighteen SNP markers constituted group 3, which could identify two genetic variants among the strains, rather than within a single strain (S1C and Supplementary Figure 1C). At the SNP marker Pb_8504, biallelic variants T/C were detected, revealing an uneven distribution of data points (Supplementary Figure 1C). This pattern indicates that one of the two alleles (T) is predominant at the Pb_8504 locus. Group 4, comprising 17 SNP markers, two homogeneous clusters were identified, and one sample (field strain AB11) was located between the two clusters and showed mixed KASP allele calls (Table 2, Supplementary Figure 1E, and Figures 4A,B). Notably, the AB11 field strain consistently appeared in the heterogeneous cluster, indicating genetic heterogeneity at these SNP loci, and suggesting that these markers can detect both heterogeneity within AB11 and two homogeneous allelic patterns among SPIs derived from this strain. To validate this, SNP markers Pb_5366 and Pb_6841 were used to genotype 20 SPIs, alongside the progenitor AB11 strain (DNA extracted from the same gall used for SPI generation) and three negative controls (one distilled water and two uninoculated healthy root samples). Genotyping revealed nine SPIs in one homogeneous cluster, ten in the other homogeneous cluster, and one heterogeneous SPI, similar to the AB11 strain’s profile (Figures 4C,D). These findings demonstrate that the SPI-generation approach is effective, as the majority of SPIs (95%) derived from the heterogeneous AB11 progenitor exhibited homogeneous genotypes at the tested loci.

**TABLE 2:** The analyzed KASP SNP markers.

Plot characteristics	No. of SNP markers	Name of SNP markers
No pattern	10	Pb_3704, Pb_3720, Pb_4665, Pb_5057, Pb_5384, Pb_7812, Pb_8417, Pb_8597, Pb_8781,Pb_9643
One genotype	15	Pb_0406, Pb_2349, Pb_2841, Pb_3855, Pb_3915, Pb_4002, Pb_5134, Pb_5907, Pb_7110, Pb_7186, Pb_7333, Pb_7986, Pb_8423, Pb_8910, Pb_9012
Two genotypes	18	Pb_0344, Pb_0825, Pb_1027, Pb_1142, Pb_1998, Pb_1893, Pb_5066, Pb_5786, Pb_6019, Pb_7302, Pb_8049, Pb_8504, Pb_8735, Pb_9016
		Pb_2242, Pb_6372, Pb_9638, Pb_9197
Three genotypes	17	Pb_2054, Pb_2395, Pb_3025, Pb_3321, Pb_3358, Pb_3387, Pb_3969, Pb_4152, Pb_4200, Pb_5347, Pb_5366, Pb_5611, Pb_6841, Pb_7752, Pb_7994, Pb_8725, Pb_8884

A total of sixty KASP SNP markers were examined, analyzed, and subsequently categorized into four distinct groups based on their plot characteristics.

### 3.5 Genetic purity assessment of *P. brassicae* SPIs through whole-genome sequencing

To further confirm the genetic purity of the SPIs, whole-genome sequencing was performed on two progenitor field strains, AB11 and AB16, along with nine SPIs derived from each. Following short-read assembly, read mapping rate, genome coverage, and sequencing depth were assessed to evaluate sequencing and alignment quality. Since DNA samples were prepared from pathogen spores purified from galls, 90% of the samples exhibited read mapping rates > 80% (Supplementary Figure 2). Consequently, the average contig-wise genome coverage exceeded 90% (Supplementary Figure 3), with a mean sequencing depth > 40 × (Supplementary Figure 4). Together, these metrics indicate uniform coverage across the pathogen genome, providing confidence for subsequent analyses of genetic diversity.

**FIGURE 5:**
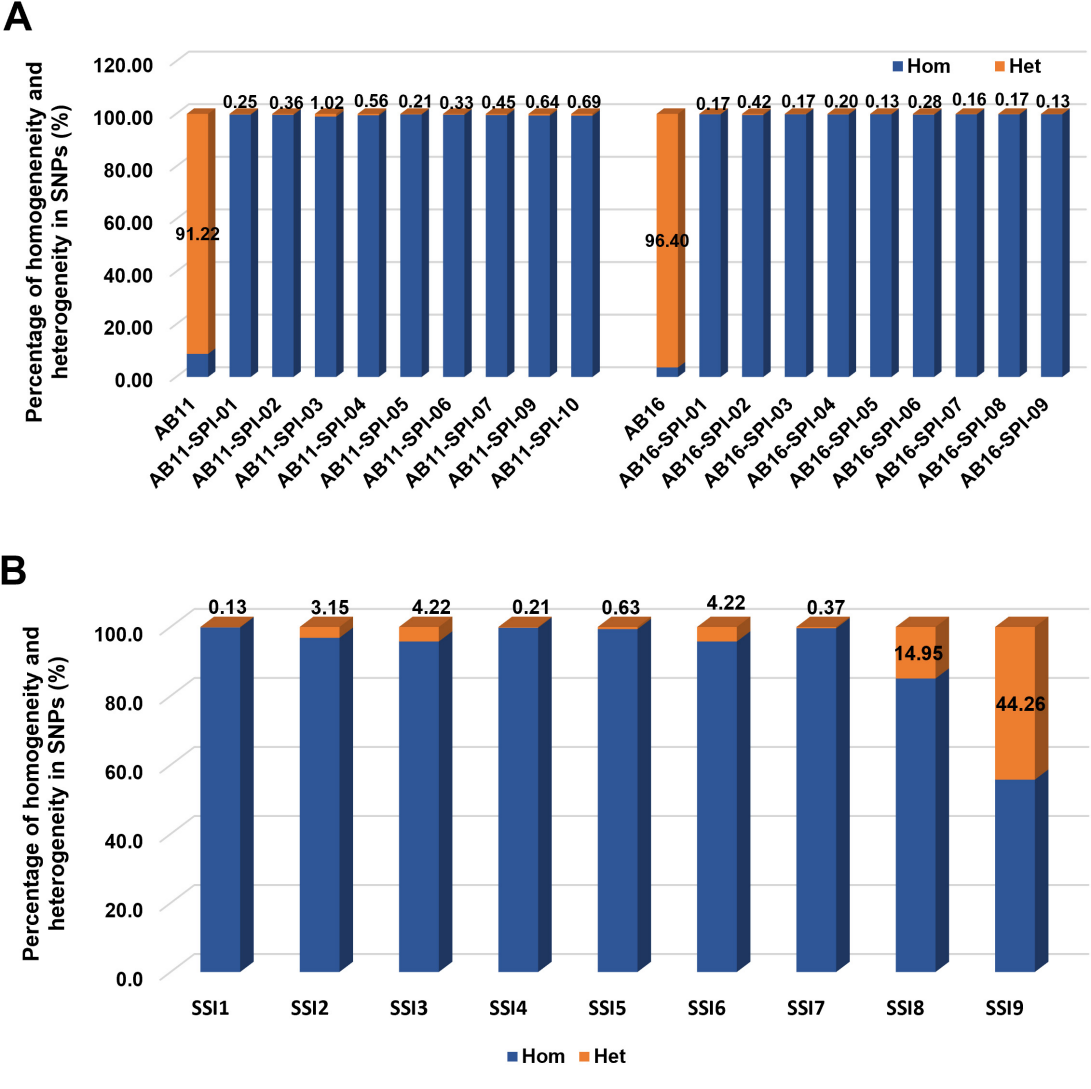
SNP heterogeneity analysis. **(A)** The SNPs of strains AB11 and AB16 and nine of their single-protoplast isolates (SPIs) generated from each were called. The homogeneous and heterogeneous SNPs were countered for the ratio calculation. **(B)** The sequences of single-spore isolates (SSIs) were downloaded from Genome-NCBI-NLM (nih.gov). The ratio of homogeneous and heterogeneous SNPs was calculated. “Hom” (homogeneous alleles) is depicted in blue, and “Het” (heterogeneous alleles) is represented in orange.

Genome-wide SNPs were subsequently identified, and their genomic distribution and heterogeneity were analyzed. The progenitor strains showed substantial heterogeneity, with heterogeneous allele rates of 91.2% for AB11 and 96.4% for AB16. In contrast, the SPIs displayed remarkably lower heterogeneity rates below 1% (Figure 5A).

SSIs are usually considered genetically pure isolates; however, this has not been experimentally confirmed. We downloaded the raw DNA sequence data of nine SSIs (Sedaghatkish et al., 2019) from the NCBI Genome database (nih.gov) and analyzed them following the same procedure used for SPIs. While four out of nine SSIs proved nearly pure homogeneous with heterogeneous allele < 1%, the other five SSIs exhibited heterogeneity rates higher than our SPIs, notably SSI8 and SSI9 reaching 14.95 and 44.26%, respectively (Figure 5B). This confirmed that the genetic purity of SPIs was comparable to that of SSIs.

### 3.6 Multiple races of *P. brassicae* identified from single clubroot galls

The races of two field strains, AB11 and AB16, as well as their corresponding SPIs, were determined using a set of canola SGLs carrying single clubroot resistance genes. Based on gene-for-gene theory and the reactions observed on those resistance lines, the field-collected strain AB11 was classified as race *Avr5-8* (Zhang et al., 2026). Interestingly, none of its nine SPIs matched this race. Instead, the SPIs were distributed across four races: three SPIs were classified as *avr1-3-5-8-9-10*, two as *Avr8*, two as *Avr8-9*, and two as *Avr3-8-9*. Notably, all SGLs were susceptible to isolates AB11-SPI-03, AB11-SPI-05, and AB11-SPI-09, which exhibited a highly aggressive virulence race, *avr1-3-5-8-9-10*, posing a significant threat to canola production unless additional resistance genes are identified. The other field collected strain, AB16, was classified as race *Avr5-10* (Zhang et al., 2026). Among its nine SPIs, three races were identified: one SPI was classified as *Avr10*, seven as *Avr5-10*, and one as *Avr5-9-10*. Race *Avr5-10*, consistent with the progenitor strain AB16, was the most prevalent (Table 3). Among the eighteen SPIs derived from both strains, the frequency of *Arv1*, *Avr3*, *Avr5*, *Avr8*, *Avr9* and *Avr10* was 0.0, 11.1, 44.4, 33.3, 27.8, and 50.0%, respectively, with *Avr5* and *Avr10* being the most prevalent *Avr* genes among the SPIs (Table 4).

**TABLE 3:** Race characterization of *P. brassicae* single protoplast-derived isolates.

Progenitor strains	SPIs derived from the progenitor strain	Races	No. of SPIs with the same race	Freq. of the races within SPIs
AB11	-	*Avr5-8*	-	-
	AB11-SPI-03, AB11-SPI-05, AB11-SPI-09	*avr1-3-5-8-9-10*	3	33.3%
	AB11-SPI-04, AB11-SPI-06	*Avr8*	2	22.2%
	AB11-SPI-07, AB11-SPI-10	*Avr8-9*	2	22.2%
	AB11-SPI-01, AB11-SPI-02	*Avr3-8-9*	2	22.2%
AB16	-	*Avr5-10*	-	-
	AB16-SPI-06	*Avr10*	1	11.1%
	AB16-SPI-01, AB16-SPI-02, AB16-SPI-03, AB16-SPI-04, AB16-SPI-05, AB16-SPI-07, AB16-SPI-09	*Avr5-10*	7	77.8%
	AB16-SPI-08	*Avr5-9-10*	1	11.1%

Two field-collected strains AB11 and AB16 were selected for producing single protoplast isolates (SPIs) to determine races of the SPIs.

**TABLE 4:** Frequencies of avirulence (*Avr*) genes among *P. brassicae* single protoplast isolates (SPIs) derived from AB11 and AB16.

*Avr* genes	No. of isolates	Freq. of *Avr* genes
*Avr1*	0	0.0%
*Avr3*	2	11.1%
*Avr5*	8	44.4%
*Avr8*	6	33.3%
*Avr9*	5	27.8%
*Avr10*	9	50.0%
No of isolates identified	18	-

*Avr* genes were characterized in 18 sequenced SPIs (Figure 3) using a set of *Brassica napus* single-gene lines (SGLs) each carrying a single clubroot resistance (*CR*) gene (*Rcr1*) or locus referred as *Rcr3*, *Rcr5*, *Rcr8*, *Rcr9*, or *Rcr10*.

## 4 Discussion

Purification of the clubroot pathogen, *P. brassicae*, is crucial for accurately studying its biology, enabling the development of targeted control measures and resistant crop varieties. Traditionally, researchers isolate single resting spores to infect *Brassica* seedlings, generating SSIs to produce genetically pure *P. brassicae* populations, as the pathogen cannot survive or proliferate *in vitro*. Although this method was developed decades ago and efforts have been made to enhance its infection success rate, it yields a low and inconsistent success rate, complicating precise race characterization of *P. brassicae* populations and hindering effective clubroot resistance breeding (Buczacki, 1977; Tinggal and Webster, 1981; Jones et al., 1982; Scott, 1985; Xue et al., 2008; Askarian et al., 2021b; Lv et al., 2021). In this study, we developed a novel approach to obtain genetically near-pure *P. brassicae* by inoculating DHW seedling roots with resting spores isolated from single clubroot-infected root protoplasts—plant cells with their cell walls removed. Compared to single spores used for SSI generation, the significantly higher number of resting spores (1,000–9,000) released from single protoplasts for SPI generation (Supplementary Figure 1E and Supplementary Table 1), ensured higher infection success rates ranging from 77.8 to 100% in Sunshine Mix #3 (Table 1). The variation in SPI infection success rates is strain-dependent, aligning with prior findings by Diederichsen et al. (2016). To achieve high infection success and maintaining genetic uniformity, the timing of protoplast isolation was a critical factor. Five wpi represents the optimal stage, as galls are fully developed and resting spores are mature, providing sufficient inoculum for SPI generation. Collecting roots earlier at 4 wpi, when spores are immature, risks lower infection efficiency and incomplete representation of the pathogen within protoplasts. Conversely, harvesting roots beyond 5 wpi increases the likelihood of gall decay and resting spore release, which can introduce variability into the inoculum during protoplast isolation. Timing protoplast isolation at 5 wpi thus ensures a practical and reproducible approach, maximizing infection success while maintaining the genetic uniformity of single-protoplast-derived isolates.

In this study, bi-allelic SNP markers revealed that 95% of 20 SPIs derived from strain AB11 were homogeneous (Figures 4C,D), providing indirect evidence for a “one-cell-one-genotype” dominance in *P. brassicae*. While previous studies reported that multiple primary and secondary plasmodia can be observed within the same root cells (Tu, 2018), the development of a secondary plasmodium is a resource-intensive process. Under nutrient-limited conditions within the host, competitive interactions among coinfecting individuals may occur (Antagonism, 2003; Hibbing et al., 2010; Baishya and Wakeman, 2019). Such intra-host competition may favor the proliferation of a single secondary zoospore or a dominant plasmodium, ultimately resulting in effectively colonization of the cortical cell. This natural intracellular filtering mechanism explains why protoplast-derived inoculum provides a level of uniformity that is difficult to achieve with mixed field populations. The 5% of heterogeneous SPIs observed may stem from free spore contamination during isolation or rare instances of “co-dominant” infections, where distinct, genetically diverse secondary zoospores successfully cohabitated and matured within the same root cortical cell, or even less frequently from genetic variation arising during pathogen reproduction itself, as mitotic or meiotic processes could introduce novel alleles even in genetic pure populations (Askarian et al., 2021b).

Whole-genome sequencing of 18 SPIs and their progenitor strains, AB11 and AB16, demonstrated that single-protoplast isolation is a highly effective approach for generating nearly genetically pure isolates of *P. brassicae*. The heterogeneous allele rate in SPIs was significantly lower ( ≤ 1%) compared to 91.22% in the field-collected strain AB11 and 94.44% in AB16. In contrast, two of nine SSIs from a public database (Sedaghatkish et al., 2019) displayed much higher heterogeneous allele rates of 14.95 and 44.26% (Figure 3B). A direct side-by-side comparison with newly generated SSIs was not feasible because the SSIs used for comparison have undergone multiple cycles of host propagation, and because the extremely low infection success rate associated with single resting spore inoculation makes *de novo* production of SSIs for parallel sequencing impractical. To minimize potential bias arising from differences in data processing, we re-analyzed the raw sequence reads from these public SSIs using our specific SNP-calling pipeline. While we acknowledge that variations in DNA library preparation and sequencing depth across studies can introduce technical noise, the consistent application of our bioinformatics parameters confirmed that these SSIs maintain significantly higher background heterogeneity. This indicates that the high purity of SPIs is a biological reality of the protoplast-derived approach rather than an artifact of sequencing technology. Theoretically, a genetically pure isolate should exhibit zero heterogeneity; the observed ≤ 1% in our SPIs likely results from minor mutations introduced during PCR amplification, sequencing errors, or alignment biases.

Previous studies have shown that field strains of *P. brassicae* often comprise multiple pathotypes, with single-spore isolates from the same pathotype displaying diverse races (Tinggal and Webster, 1981; Askarian et al., 2021b). In this study, none of the races identified among the SPIs derived from progenitor strain AB11 matched the original progenitor race (*Avr5-8*) (Zhang et al., 2026); similarly, *Avr9* was detected in AB16-derived SPIs despite being absent in the progenitor. This discrepancy suggests a complex “masking” effect within field populations, where mixed pathotypes lead to inaccurate resistance labeling. In a mixed population, highly aggressive strains (such as the *avr1-3-5-8-9-10* SPI identified here) may exist as numerical minorities or have their phenotypic expression suppressed by non-aggressive strains through competition for host resources or induced host defenses (Vaumourin et al., 2015; Dutt et al., 2022). For example, the presence of *Avr8* in AB11 may have elicited plant defenses that prevented the detection of aggressive strains carrying *avr5*. Conversely, the failure to detect *Avr3* and *Avr9* in the mixed AB11 strain could be attributed to certain components of the mixture suppressing host defenses, as seen with *Albugo candida* in *Arabidopsis* (Cooper et al., 2008). These findings carry significant implications for disease management: the deployment of specific resistance genes (e.g., *Rcr5* or *Rcr8*) could act as a selective pressure that removes dominant, non-aggressive strains, effectively “releasing” these hidden, suppressed virulent isolates. This phenomenon may lead to the rapid breakdown of resistance in the field. By unmasking this cryptic diversity, the SPI method enables the precise assessment of genetic variation and more reliable *Avr* gene assignment, allowing breeders to account for “hidden” threats before widespread resistance deployment.

Understanding the distribution and frequency of *Avr* genes provides insights into their evolutionary dynamics, enabling proactive monitoring of virulence shifts in agricultural fields and informing the strategic deployment of resistance genes. This approach can be implemented through three main steps: (1) field collection of galls, (2) generation of SPIs, and (3) *Avr* gene assignment through phenotypic assessment using a set of clubroot resistance lines (e.g., SGLs used in this study). Notably, the detection of the highly aggressive race *avr1-3-5-8-9-10* detected in three AB11 SPIs (e.g., AB11-SPI-05) (Table 3), indicates the emergence of a highly virulent race within the year of sample collection. This finding underscores an urgent need to identify and deploy novel *CR* genes, as the increasing prevalence of such races could overcome existing host resistance and pose a challenge to clubroot management in canola (Strelkov et al., 2018). In contrast, the race *Avr*5-10, detected at a frequency of 77.8% among AB16 SPIs, appears to be the dominant race within that strain (Table 3). These results demonstrate that generating SPIs provides a robust method for accurately assessing the race composition of *P. brassicae* populations, a critical step for identifying new sources of genetic resistance, predicting race dynamics in future growing seasons, and guiding the development of resistant canola cultivars.

Moreover, integrating two or more *CR* genes into a single cultivar represents a promising strategy to enhance durability and mitigate the impact of clubroot on canola production, while reducing the risk of resistance breakdown (Hirani et al., 2018; Botero-Ramirez et al., 2024). The SPI generation method significantly facilitates this process by enabling precise identification of virulence races. Based on characterization of the races of SPIs, researchers can strategically combine *CR* genes such as *Rcr5* and *Rcr10*, to target prevalent *Avr* genes like *Avr5* and *Avr10*, creating cultivars with resistance targeting multiple *Avr* genes (Table 4). This approach not only addresses the immediate threat posed by highly virulent races but also proactively strengthens canola against evolving *P. brassicae* populations. The synergy between SPI-based race identification and *CR* gene stacking offers a powerful framework for sustainable clubroot management. By resolving the genetic complexity of *P. brassicae* populations, SPIs provide a foundation for tailored breeding strategies that align with the specific race dynamics of a given region.

## Data Availability

All data presented in this study are included in this article/Supplementary material. The raw data supporting the conclusions of this article have been deposited in the NCBI BioProject repository with accession number PRJNA1390729.

## Footnotes

1
https://www.dnastar.com/manuals/seqman-ngen/17.1/en/topic/part-a-setting-up-the-assembly-in-seqman-ngen

## Supplementary material

Supplementary Table 1 The number of resting spores in single infected root protoplasts was determined. Resting spores from 12 single protoplasts of *P. brassicae* strain AB11 were released in 100 μL of distilled water. Spores in 1 μL from the 100 μL spore suspension were counted on a hemocytometer, with three replicates performed for accuracy.

Supplementary Table 2 Genome sequences of *P. brassicae* in the public domain used for SNP discovery.

Supplementary Table 3 Flanking sequences of bi-allelic SNPs in *P. brassicae* and the corresponding KASP marker sequences. Based on the flanking sequences of 60 bi-allelic SNPs, derived from 19 distinct pathogen strains and the reference genome (Table 2), KASP marker sequences include two allele-specific primers and one common primer were designed for each SNP.

Supplementary Table 4 A small *P. brassicae* population was used to investigate single nucleotide polymorphisms (SNPs) through the KASP assay. Samples No. 1∼3, negative controls; No. 4∼8, single-protoplast isolates of *P. Brassicae* derived from AB25 strain; No. 9∼24, thirteen strains collected from Alberta fields and three strains collected from fields in Saskatchewan, named with the prefix “AB” or “SK” respectively.
