# Supplementary material for: A novel and efficient method for producing high-purity single protoplast-derived isolates of Plasmodiophora brassicae
Source: Front Microbiol. 2026 Apr 13;17:1789807. doi: 10.3389/fmicb.2026.1789807 (PMC13111542; doi:10.3389/fmicb.2026.1789807)
Supplement: Supplementary file 1 [file Presentation_1.pptx]

## Slide 1
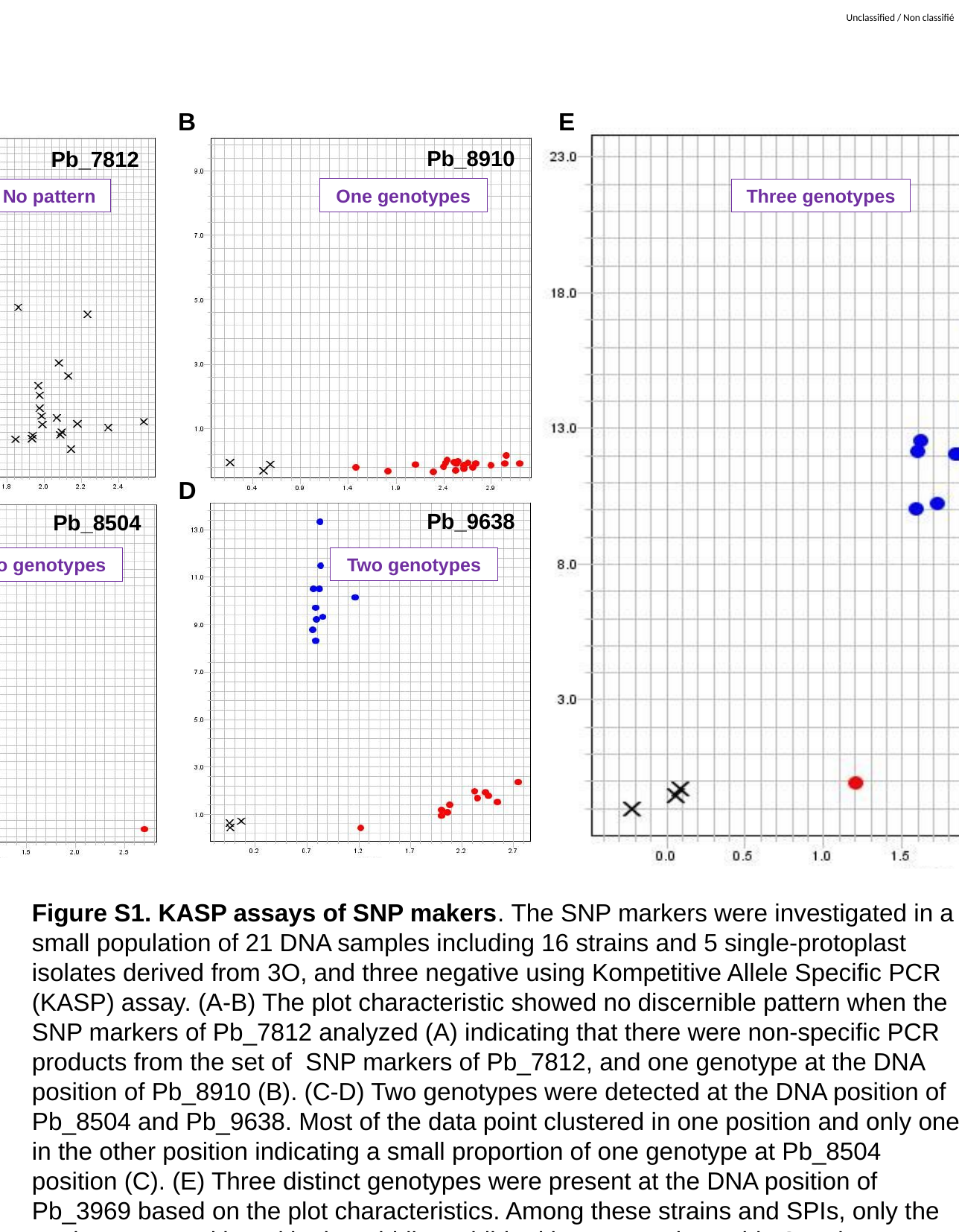

A
B
E
Pb_3969
Pb_8910
Pb_7812
One genotypes
No pattern
Three genotypes
C
D
Pb_9638
Pb_8504
Two genotypes
Two genotypes
AB11
Figure S1. KASP assays of SNP makers. The SNP markers were investigated in a small population of 21 DNA samples including 16 strains and 5 single-protoplast isolates derived from 3O, and three negative using Kompetitive Allele Specific PCR (KASP) assay. (A-B) The plot characteristic showed no discernible pattern when the SNP markers of Pb_7812 analyzed (A) indicating that there were non-specific PCR products from the set of SNP markers of Pb_7812, and one genotype at the DNA position of Pb_8910 (B). (C-D) Two genotypes were detected at the DNA position of Pb_8504 and Pb_9638. Most of the data point clustered in one position and only one in the other position indicating a small proportion of one genotype at Pb_8504 position (C). (E) Three distinct genotypes were present at the DNA position of Pb_3969 based on the plot characteristics. Among these strains and SPIs, only the strain AB11, positioned in the middle, exhibited heterogeneity at this SNP locus

## Slide 2
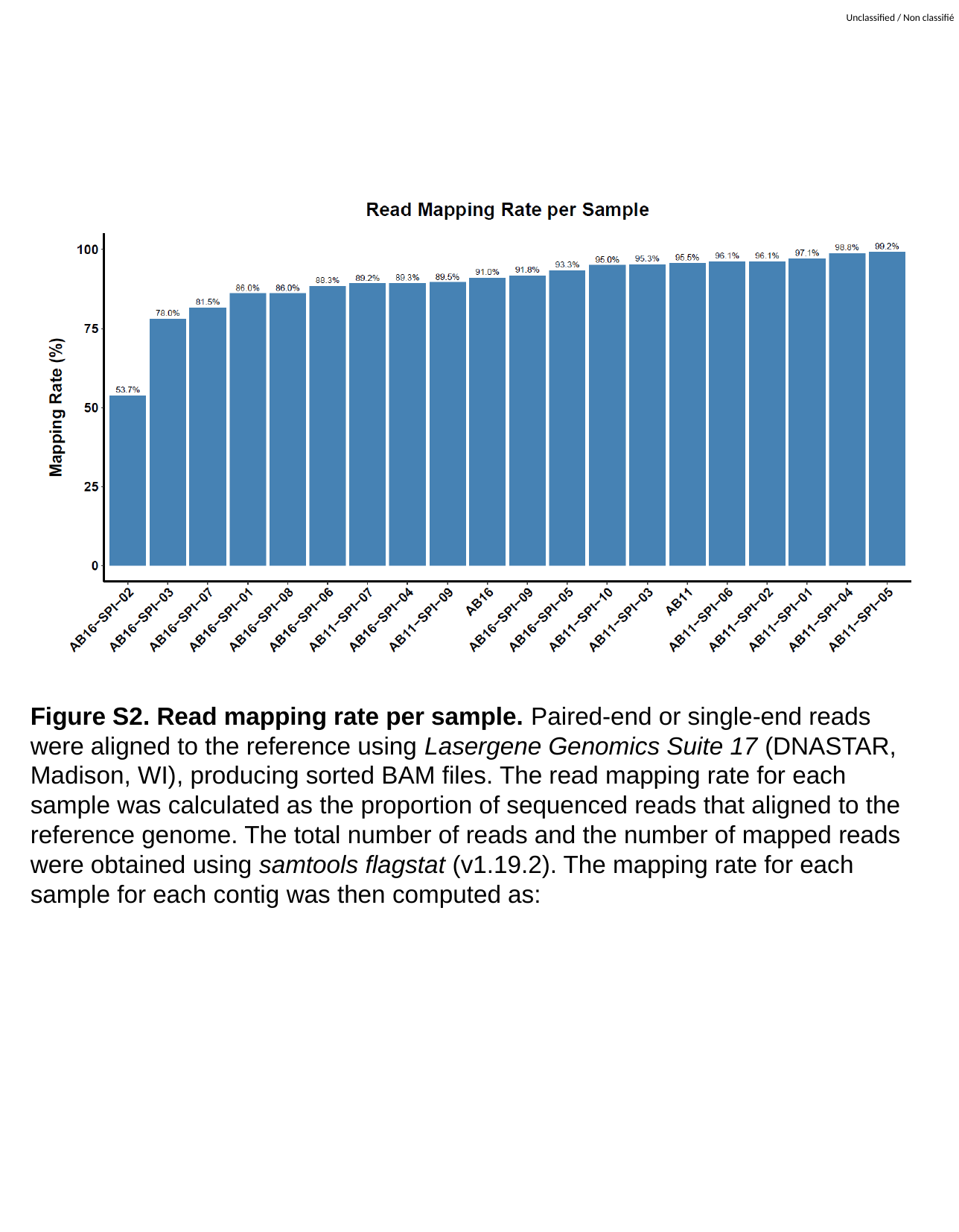

## Slide 3
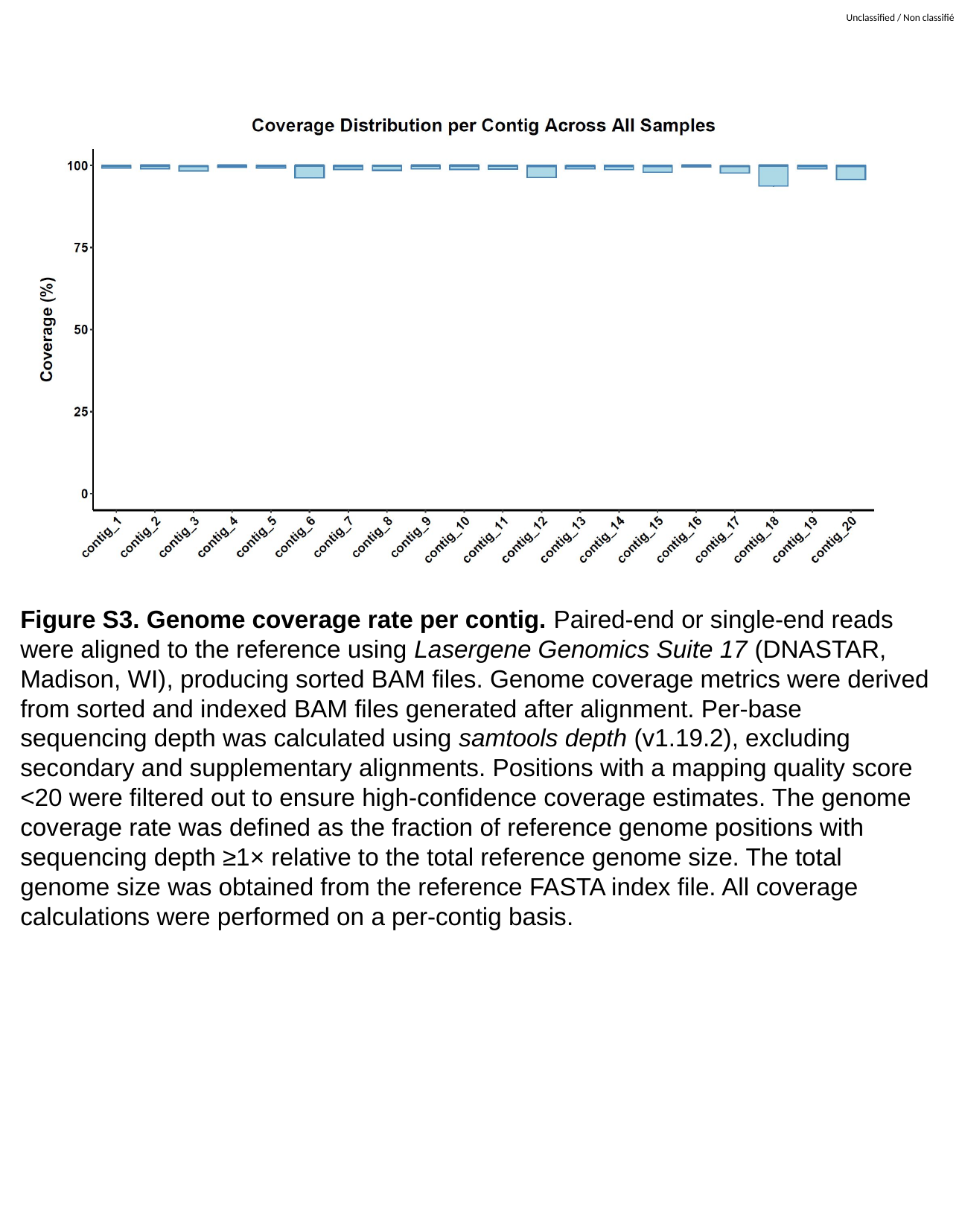

Figure S3. Genome coverage rate per contig. Paired-end or single-end reads were aligned to the reference using Lasergene Genomics Suite 17 (DNASTAR, Madison, WI), producing sorted BAM files. Genome coverage metrics were derived from sorted and indexed BAM files generated after alignment. Per-base sequencing depth was calculated using samtools depth (v1.19.2), excluding secondary and supplementary alignments. Positions with a mapping quality score <20 were filtered out to ensure high-confidence coverage estimates. The genome coverage rate was defined as the fraction of reference genome positions with sequencing depth ≥1× relative to the total reference genome size. The total genome size was obtained from the reference FASTA index file. All coverage calculations were performed on a per-contig basis.

## Slide 4
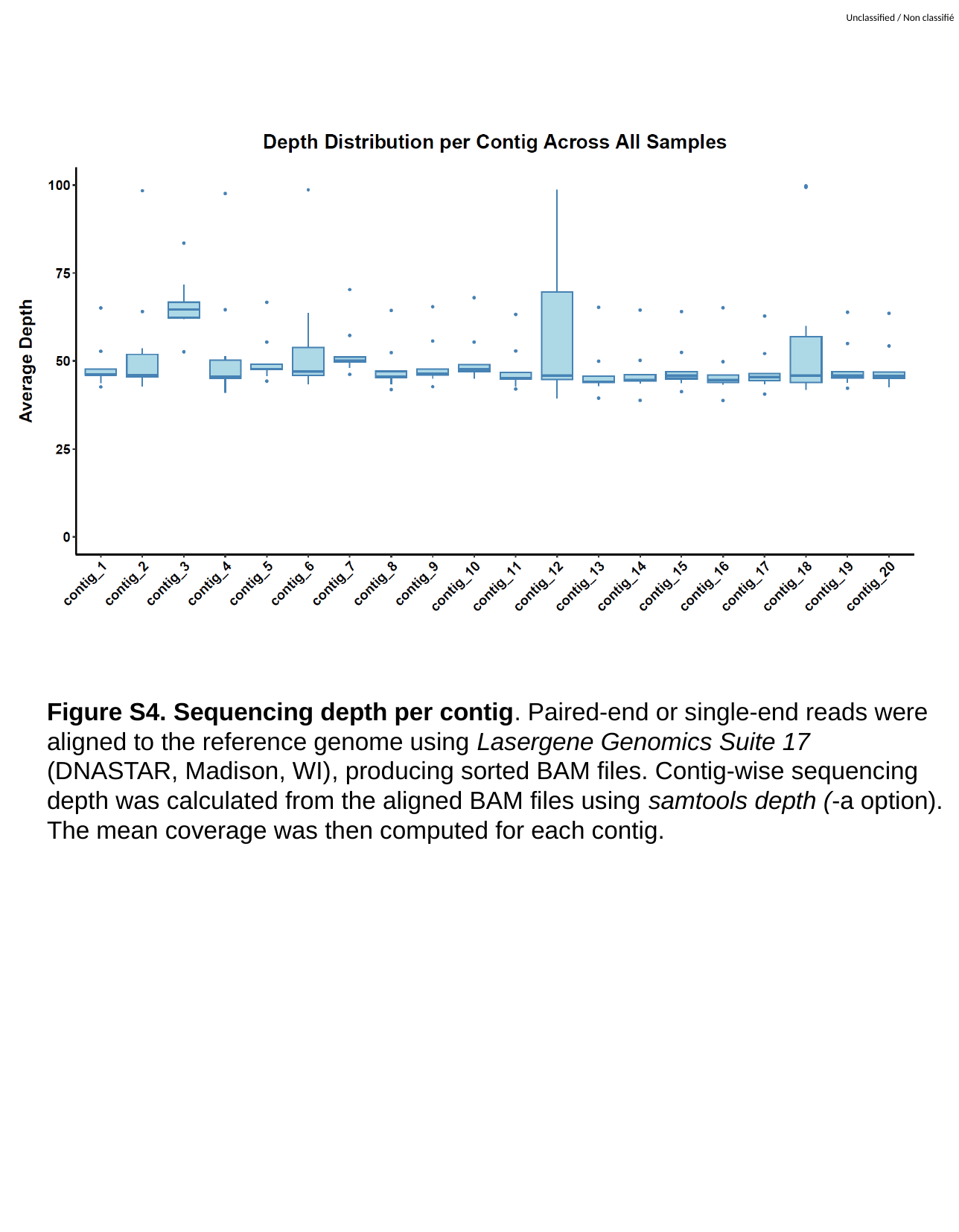

Figure S4. Sequencing depth per contig. Paired-end or single-end reads were aligned to the reference genome using Lasergene Genomics Suite 17 (DNASTAR, Madison, WI), producing sorted BAM files. Contig-wise sequencing depth was calculated from the aligned BAM files using samtools depth (-a option). The mean coverage was then computed for each contig.
